# Non-Pharmacological Approaches to Addressing Overweight and Obesity in Children and Adolescents with Mental Illness: A Scoping Review of Quantitative and Qualitative Evidence

**DOI:** 10.3390/bs16010056

**Published:** 2025-12-29

**Authors:** Annika Nordkamp, Grete Teilmann, Martin Jorsal, Justina Petersen, Julie Midtgaard

**Affiliations:** 1 Mental Health Centre Glostrup, Copenhagen University Hospital—Mental Health Services CPH, Centre for Applied Research in Mental Health Care (CARMEN), 2600 Copenhagen, Denmark; 2 Child and Adolescent Mental Health Center, Copenhagen University Hospital—Mental Health Services CPH, 2900 Copenhagen, Denmark; 3 Department of Clinical Medicine, Faculty of Health and Medical Sciences, University of Copenhagen, 2200 Copenhagen N, Denmark; 4 Department of Pediatrics, North Zealand Hospital, 3400 Hillerød, Denmark; 5 The Danish Concussion Center, 2300 Copenhagen S, Denmark

**Keywords:** children, adolescent, mental health, mental illness, psychiatry, health promotion, overweight, obesity

## Abstract

Children and adolescents with mental illness are at increased risk of developing overweight and obesity, a relationship that is complex, bidirectional, and often exacerbated by the weight-related side effects of psychotropic medications. This review addressed the research question: How are overweight and obesity addressed in children and adolescents with mental illness? Following JBI and PRISMA-ScR guidelines, a systematic search of PubMed, CINAHL, EMBASE, and PsycINFO was conducted, including studies in English or Scandinavian languages, across all designs, that focused on non-pharmacological approaches for this population aged 0–19 years. The search was completed in May 2025. Nine studies met the inclusion criteria, comprising four qualitative studies, four cohort studies, and one cross-sectional study. Based on inductive content analysis, three overarching themes were developed: approaches to weight and health, showing a predominant focus on individual lifestyle modifications; roles, resources, and prioritization, reflecting how constrained resources influence healthcare professionals’ decisions; and critical repercussions and future directions, highlighting the consequences for children, adolescents, and their families. Overall, interventions mainly target behavior change, with limited attention to structural or systemic factors. These findings underscore the need for tailored guidance and clear clinical strategies to support healthcare professionals and families in addressing weight-related issues in child and adolescent mental health care.

## 1. Introduction

Children and adolescents with mental illness (CAMIs) represent a vulnerable group facing an elevated risk of developing overweight and obesity (Chao et al., 2019; Khan et al., 2016; Li et al., 2020). A cross-sectional study (Muskens et al., 2025) in the Netherlands investigating the prevalence of somatic comorbidities in children and adolescents with psychiatric disorders identified obesity as a significant physical challenge and found that 20% were classified as overweight and 12% exhibited signs of obesity, highlighting the increased risk of overweight in this population. In a systematic review (Kokka et al., 2023) it was found that anxiety and depression are associated with the development of both overweight and obesity in children and adolescents.

The co-occurrence of overweight and mental illness poses a dual challenge, as both conditions are independently associated with stigma, teasing, and bullying (Kaushik et al., 2016; Rankin et al., 2016). Children with obesity often experience a range of psychological issues, including stress, low self-esteem, depression, body dissatisfaction, and social difficulties (Newson & Abayomi, 2025; Vander Wal & Mitchell, 2011). These psychological challenges can exacerbate the development of obesity, leading to a vicious cycle that significantly impacts their overall well-being and quality of life (Hupparage et al., 2023). The psychosocial effects of childhood obesity are a cause for concern, given their potential to persist and negatively influence emotional health throughout adulthood (Gallagher et al., 2023).

In a systematic review, Sanders et al. (2015) found that children and adolescents with overweight or obesity experience heightened cardio-metabolic risks, including non-alcoholic fatty liver disease, as well as poor psychological outcomes such as depression, low self-esteem, and reduced health-related quality of life—compared to their normal-weight peers.

The increasing use of psychotropic medications in this population (Harrison et al., 2012) has been associated with adverse metabolic outcomes, such as weight gain, hyperlipidemia, and insulin resistance (Solmi et al., 2020). For instance, atypical antipsychotics, alternatively referred to as second-generation antipsychotics, are medications used in the management of severe mental illnesses like schizophrenia and bipolar disorder. In contrast to first-generation antipsychotics, second-generation agents exhibit a lower risk of extrapyramidal symptoms but a greater likelihood of metabolic adverse events, such as weight gain and insulin resistance (Correll & Kratochvil, 2008; Muench & Hamer, 2010). Patel et al. (2007) found that children hospitalized and prescribed atypical antipsychotics experienced overweight rates three times higher than the national average in the United States. The severe impact of antipsychotic medications on pediatric patients is also demonstrated in a study (Correll et al., 2009), where drug-naïve children and adolescents gained over 4.5 kg after just 12 weeks on second-generation antipsychotics such as aripiprazole, olanzapine, quetiapine, and risperidone, regardless of age, pubertal status, ethnicity, or gender. Similarly, Schwartz et al. (2016) reported that long-term use of antidepressants in children and adolescents is associated with significant weight gain over time, with an average weight gain of 2.10 kg by age 18.

Both obesity and severe mental illness independently diminish quality of life and are linked to increased disability, morbidity, and mortality (Avila et al., 2015). When these conditions occur together, the negative health outcomes are exacerbated (Sanders et al., 2015), underscoring the importance of addressing (i.e., actively recognizing and responding to a specific issue or need through communication, assessment or intervention) these issues. Healthcare professionals in clinical settings are in a unique position to identify, prevent, or address health problems in ways that ensure patients and their families feel seen and understood. This potential is reflected in the ‘Make Every Contact Count’ approach (Phillips, 2019), which encourages professionals to use routine interactions with patients as opportunities to provide health promotion.

While Nyboe et al. (2019) reviewed non-pharmacological lifestyle interventions in young patients (aged 13–45 years) focusing on effectiveness, less is known about non-pharmacological clinical and organizational approaches used to address overweight and obesity in child and adolescent mental health care.

Mapping ways to address overweight and obesity in child and adolescent mental health care is crucial for tailoring interventions that are sensitive to mental health needs, avoiding potential harms such as stigmatization or exacerbation of psychological distress, and fostering sustainable, health-promoting behaviors. Therefore, the aim of this scoping review was to explore the existing evidence on non-pharmacological approaches to addressing overweight and obesity in children and adolescents with mental illness. The specific objectives were to identify: (1) types of interventions, settings, and outcomes, and (2) gaps in the current research.

## 2. Materials and Methods

A scoping review approach was chosen in this study because ways to address co-occurring mental illness and overweight in child and adolescent mental health care is a research area where concepts are still evolving and a wide range of study designs is expected (Arksey & O’Malley, 2005; Levac et al., 2010; Peters et al., 2020b). Joanna Briggs Institute’s review manual for scoping reviews (Peters et al., 2020a) and the PRISMA Extension for Scoping Reviews (PRISMA-ScR) (Tricco et al., 2018) were followed (see Supplementary Material). The review addressed the research question: How are overweight and obesity addressed in children and adolescents with mental illness? To guide the study selection, the Population, Concept and Context (PCC) framework (24) was used (see Table 1). The protocol was preregistered on Open Science Framework (OSF) on 19 April 2024, before commencing the study: https://osf.io/t86f7/.

### 2.1. Search Strategy

The search strategy was developed in collaboration with an information specialist and performed in PubMed, CINAHL, EMBASE, and PsycINFO. In addition, a search was conducted for grey literature, encompassing guidelines and reports concerning overweight and obesity in CAMIs, and guidelines and reports on antipsychotic-induced weight gain in children and adolescents; however, no relevant guidelines were found. The literature search was uploaded into the Covidence systematic review software, Veritas Health Innovation, Melbourne, Australia. Available at www.covidence.org for screening.

### 2.2. Search Teams

The search combined both free-text terms and Medical Subject Headings (MeSH) related to mental disorders (e.g., “ADHD”, “depression”, “bipolar disorder”, “schizophrenia”, “autism”, “personality disorder”, and the broader terms “mental illness” and “mental disorder*”) with terms related to overweight and obesity (e.g., “pediatric obesity”, “adolescent obesity”). The full search strategy is available upon request from the corresponding author.

### 2.3. Screening and Selection

Two authors (A.N. and M.J.) dually screened titles and abstracts based on inclusion and exclusion criteria. Full-text screening was performed independently by three authors (A.N., M.J. and J.P.) to decide which articles should be included in the review. Any disagreements were thoroughly discussed until a consensus was reached.

### 2.4. Data Extraction

A draft data extraction form to capture the characteristics of the included studies was developed specifically for our topic. Data were extracted independently by two authors, A.N. and J.P., from the first three studies and were subsequently discussed internally between A.N., J.P., and M.J. until consensus was reached on what data should be extracted, after which adjustments were made. Extracted data included study characteristics, participant and intervention characteristics, and outcomes (see Table A1).

### 2.5. Qualitative Content Analysis

As suggested by Pollock et al. (2023), we based our analysis on Elo and Kyngäs’s (2008) three phases of qualitative content analysis: (1) preparation, (2) organizing, and (3) reporting. The method is suitable across various evidence sources or study designs within a scoping review and is not restricted to primary qualitative studies (Pollock et al., 2023). Given the anticipated limited knowledge in the field, we employed an inductive approach, deriving categories from the data. One author (A. N.) performed iterative reading, open coding, category development, and abstraction. Categories were subsequently merged and organized into overarching themes in collaboration between A.N. and J.M.

## 3. Results

**Figure 1:**
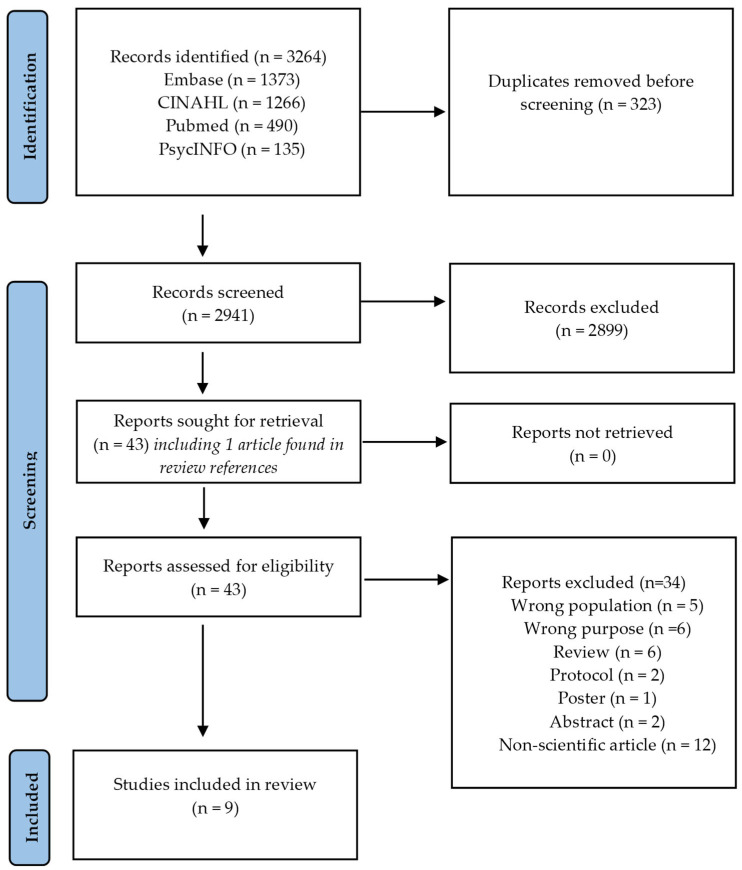
Flowchart illustrating literature search and selection.

We identified 3264 records, of which 2899 were excluded. Following this, 43 full-text articles underwent eligibility screening, with 34 being excluded. Consequently, 9 peer-reviewed publications were included in the final review (see Figure 1).

### 3.1. Characteristics of Studies

The studies originated from two countries (USA and Canada), including four qualitative studies (Bourassa et al., 2017; Jachyra et al., 2018, 2019; Walker et al., 2020), four cohort studies (Dettmer et al., 2021; Espinoza et al., 2021; Killian et al., 2022; Taylor et al., 2019) and one cross-sectional study (Wykes et al., 2022), and was conducted across a range of settings.

### 3.2. Summary of Results

**Figure 2:**
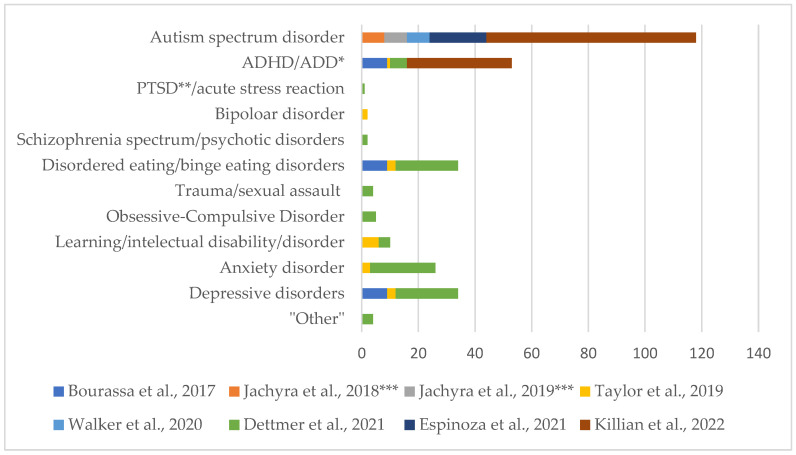
Number and distribution of represented diagnoses. 42 of the children and adolescents had more than one diagnosis/major mental health concern. * ADD Attention Deficit Disorder; ** PTSD Post Traumatic Stress Disorder; *** the same 8 adolescents are involved in both studies by Jachyra et al. Data presented in this figure are based on previously published studies from the current review, including Bourassa et al. (2017); Jachyra et al. (2018, 2019); Taylor et al. (2019); Walker et al. (2020); Dettmer et al. (2021); Espinoza et al. (2021); and Killian et al. (2022). Colors correspond to individual studies, as indicated in the figure legend.

A total of 181 children and adolescents (aged 2–18) with 12 different diagnoses (see Figure 2), 28 caregivers and 121 health care professionals (HCPs) were represented in the studies. Two studies (Jachyra et al., 2018, 2019) were conducted by the same author, with the same group of young participants in both studies. Three studies (Bourassa et al., 2017; Dettmer et al., 2021; Espinoza et al., 2021) included income and educational level. Killian et al. (2022) noted that those with insurance were more likely to attend the follow-up than participants without. Collectively, the three studies (Bourassa et al., 2017; Dettmer et al., 2021; Espinoza et al., 2021) revealed significant economic and educational challenges among families with children with mental illness, characterized by high unemployment rates (64.7%) (Bourassa et al., 2017), low educational attainment (only 39% holding a college degree) (Dettmer et al., 2021), and a majority living below 200% of the federal poverty level (80.4%) (Espinoza et al., 2021).

### 3.3. Content Analysis

The inductive content analysis of the studies resulted in 14 codes, from which three categories were derived: approaches to weight and health; roles, resources and prioritization; and critical repercussions and future directions (see Table 2).

#### 3.3.1. Approaches to Weight and Health

A recurring feature observed across all studies (Bourassa et al., 2017; Dettmer et al., 2021; Espinoza et al., 2021; Jachyra et al., 2018, 2019; Killian et al., 2022; Taylor et al., 2019; Walker et al., 2020; Wykes et al., 2022) was a consistent emphasis on individual lifestyle modifications, particularly focused on diet and exercise, aimed at achieving weight reduction among CAMIs. This is specifically reflected in the four intervention studies (Dettmer et al., 2021; Espinoza et al., 2021; Killian et al., 2022; Taylor et al., 2019), which all used changes in BMI as their primary outcome measure. The study by Taylor et al. (2019), conducted in an inpatient setting, distinguished itself through a more structural approach. Instead of directly influencing individuals’ motivation or attitudes, the study focused on modifying the environment by prohibiting external food and securing food areas with passcodes.

Studies reporting the perspectives of HCPs showed that HCPs tended to place the burden of weight gain squarely on the individual (Jachyra et al., 2018, 2019). Jachyra et al. (2018) identified that HCPs emphasized personal responsibility in weight management, while Jachyra et al. (2019) further confirmed that HCPs frequently attribute weight problems to individual decisions.

Bourassa et al. (2017) showed that some children and adolescents may equate thinness with health, also reflecting a rather simplistic understanding of body weight and health. In contrast, the children and adolescents in the study by Walker et al. (2020) viewed weight gain as a normal part of growth, not necessarily as a negative indicator of health.

Espinoza et al. (2021) occasionally used the term “healthy weight” instead of “overweight”. Dettmer et al. (2021) specifically mentioned weight maintenance, and not solely weight loss, as a criterion for success, and the intervention focused on promoting a healthy lifestyle by increasing physical activity, fostering healthy eating habits, and developing general mental health skills, rather than solely on weight reduction.

Across all studies (Bourassa et al., 2017; Dettmer et al., 2021; Espinoza et al., 2021; Jachyra et al., 2018, 2019; Killian et al., 2022; Taylor et al., 2019; Walker et al., 2020; Wykes et al., 2022), the importance of psychosocial factors in enhancing health and well-being was acknowledged. All four intervention studies (Dettmer et al., 2021; Espinoza et al., 2021; Killian et al., 2022; Taylor et al., 2019) emphasized the intersection of mental health and weight management. This was reflected in interventions that also included therapy (Dettmer et al., 2021; Taylor et al., 2019), family involvement (Dettmer et al., 2021; Espinoza et al., 2021; Taylor et al., 2019), and were tailored with an inclusive approach (Espinoza et al., 2021; Killian et al., 2022) to meet the specific needs of the children and adolescents considering their mental health conditions and focusing on their mental well-being. However, only Dettmer et al. (2021) incorporated these aspects into their outcome measurements, indicating that the primary emphasis in weight management remains focused on changes in BMI.

#### 3.3.2. Roles, Resources and Prioritization

Studies showed that HCPs prioritized the mental health of the youth, viewing it as their primary responsibility, with weight management and overall health as secondary concerns (Bourassa et al., 2017; Jachyra et al., 2018). This prioritization was shared by parents, who focused on the youth’s physical health only after the mental illness was under control (Bourassa et al., 2017). HCPs aimed to balance between the need for antipsychotic medication and its weight gain side effects (Jachyra et al., 2018). Physical health and weight were approached cautiously alongside mental illness management, as there were concerns that the demands might be overly burdensome (Bourassa et al., 2017; Jachyra et al., 2018).

Bourassa et al. (2017) highlighted time constraints and limited training opportunities as barriers to addressing weight-related issues, with the latter also mentioned in the study by Jachyra et al. (2018). Similarly, Wykes et al. (2022) described a lack of workplace guidelines and elaborated that healthcare professionals primarily acquired their knowledge on how to address weight-related issues and advise families through internet searches (Wykes et al., 2022). Specifically, Taylor et al. (2019) highlighted challenges, but not impossibilities, pertaining to staff training in implementing and adhering to behavioral plans.

Regarding the role of the parents, Jachyra et al. (2018) described that parents and caregivers played a significant role in conversations about weight-related topics and sought to reinforce the health promotion information provided by the HCPs (Jachyra et al., 2018). Bourassa et al. noted that the accessibility of nutritious food and activities affected their ability to provide their children with a healthy lifestyle (Bourassa et al., 2017). HCPs observed that parents with mental health issues or those exhausted from caring for a child with mental health problems found it challenging to prioritize physical health (Bourassa et al., 2017).

Involvement of family members was acknowledged as beneficial in weight management interventions, yet active engagement posed challenges. Dettmer et al. (2021) described that these difficulties stemmed from personal life factors, such as financial stressors, conflicting work schedules, and transportation burdens. Similarly, Taylor et al. (2019) noted that implementing family and group therapies encountered challenges because of the limited availability of parents and family members. Espinoza et al. (2021) highlighted that families expressed appreciation for the inclusive measures implemented to facilitate their children’s participation.

#### 3.3.3. Critical Repercussions and Future Directions

Three studies (Bourassa et al., 2017; Jachyra et al., 2018; Wykes et al., 2022) described a recurring apprehension among HCPs, including the fear of offending patients, inducing stigma, or damaging the therapeutic relationship when discussing weight-related issues. Studies showed that this concern often led HCPs to approach these conversations with caution, aiming to avoid exacerbating any existing body image issues or contributing to feelings of shame.

Despite HCPs’ cautious attention to stigma, some efforts to address weight inadvertently had the opposite effect, reinforcing negative self-perceptions among children and adolescents, which is described in both studies by Jachyra et al. (2018, 2019), and reported that both children and caregivers felt shame over their inability to manage the child’s weight.

Children and adolescents expressed frustration when HCPs insisted on behavioral and nutritional changes without proper guidance (Jachyra et al., 2019). Adolescents and caregivers noted that discussions about weight frequently intensified their feelings of shame and discomfort (Jachyra et al., 2018, 2019). During clinical encounters, some children and adolescents adopted avoidance strategies when weight was discussed, while they expressed difficulty in coping with their increasing weight and changing bodies (Jachyra et al., 2019).

According to Bourassa et al. (2017), healthcare professionals expressed that weight-related discussions were more constructive and less distressing when it was established beforehand that weight and health were part of the treatment goals. Similarly, Jachyra et al. (2019) found that children and adolescents felt that being prepared for the content of the conversation made it easier to engage.

Bourassa et al. (2017) highlighted that HCPs emphasized the importance of collaborating with other medical professionals, allowing them to maintain their focus on mental health while prioritizing aspects of mental health that are interconnected with general health and habits, such as emotional eating. Finally, the importance of a good therapeutic relationship was highlighted by both children and adolescents, caregivers, and HCPs as a prerequisite for effective weight-related conversations (Jachyra et al., 2018).

## 4. Discussion

The result of this scoping review highlights a striking paucity of research on ways of addressing and non-pharmacologically managing overweight and obesity in CAMIs. This is particularly concerning given the rising number of children and adolescents in need of treatment for mental health issues (Kieling et al., 2011) and the increased risk of being or becoming overweight in this population (Förster et al., 2023).

The included studies revealed different challenges in addressing and managing overweight in CAMIs, including time constraints (Bourassa et al., 2017), limited opportunities for further training (Bourassa et al., 2017; Jachyra et al., 2018), and a lack of workplace guidelines (Wykes et al., 2022).

We uncovered a predominantly individualistic perspective towards the challenge of reducing overweight in children and adolescents with mental illness. This focus entails personal responsibility for health and behavior change, placing the burden of maintaining good health entirely on the individual (Grannell et al., 2021), and in this case, the child and their family. It attributes overweight solely to personal choices and willpower, disregarding the broader social, environmental, and genetic factors that also impact weight (Brownell et al., 2010). This perspective is also evident in wider beliefs held within the healthcare sector and society, which posit that being overweight is predominantly a result of personal decisions (Pearce et al., 2021). However, we identified one example (Taylor et al., 2019) in the literature of an alternative, more structural approach altering the environment around the individual in inpatient care (i.e., within a confined area and a defined period). The intervention involved prohibiting external food and securing food areas with passcodes, eliminating personal choice, and completely preventing adoption and maintenance of behaviors that contributed to weight gain. Given the limited research in this area, it remains unclear which strategy (i.e., individualistic and/or structural) best aligns with the preferences and needs of the target population and therefore is likely to be most effective long-term.

However, it is important to acknowledge the broader systemic and structural influences that shape health outcomes in adolescents with mental illness and co-occurring overweight or obesity. Health policy, service organizations, and the availability of trained professionals are central to enabling or constraining access to effective, integrated care (Kopelman et al., 2007; Marmot et al., 2008). Without attending to these upstream factors, interventions may risk reinforcing individual responsibility narratives while overlooking the social determinants that contribute to unequal outcomes and intervention uptake.

Despite the lack of knowledge in this area, our findings regarding the individualistic perspective are particularly concerning, given that key cognitive and psychological functions can be impaired in CAMIs. As demonstrated in a recent review and meta-analysis (East-Richard et al., 2020), significant deficits in executive functions (such as planning, impulse control, and problem-solving) as well as episodic memory (the ability to recall events and experiences) were frequently observed, regardless of the psychiatric disorder in question. This makes it more difficult for individuals to engage and accomplish the requirements of behavior change. Considering the typically low socioeconomic resources reported in this population, it is essential to develop and implement targeted support and intervention strategies that specifically address and accommodate these unique challenges.

The prevailing narrative that frames obesity as a matter of individual responsibility often unjustly places blame on those affected, leading to significant stigma associated with the condition (Puhl & Heuer, 2010). This is especially paradoxical as many of the children and adolescents who participated in the studies (Bourassa et al., 2017; Jachyra et al., 2018, 2019; Killian et al., 2022) had been prescribed medications with side effects that contributed to weight gain. These challenges faced by children and adolescents are not unique to this age group as underscored in a qualitative study by Usher et al. (2013), that showed adults experiencing weight gain due to second-generation antipsychotics struggled significantly with managing an insatiable appetite.

However, this does not imply that healthcare professionals in clinical practice cannot significantly influence behavior change. Recent research emphasizes the importance of including psychosocial and familial factors in obesity intervention programs (Halberstadt et al., 2023; Sepulveda et al., 2019), and as suggested by Phillips (2019), nurses should develop strategies for promoting health and consider how to integrate the ‘Making Every Contact Count’ approach and enhance the overall effectiveness of their practice. If strategically leveraged, it may benefit families and patients and improve overall health outcomes (Phillips, 2019; Swinburn et al., 2019).

Research suggests that interventions focused solely on individual behavior change rarely address the root causes of obesity, such as personal self-control, societal attitudes, body image dissatisfaction, and socioeconomic factors like age, gender and education (Dogbe et al., 2021; Swinburn et al., 2019). The need for a broader and more holistic approach that incorporates these multifaceted determinants in child and adolescent mental health care is essential for addressing not only the physical aspects of obesity but also the psychological and social dimensions affecting young people (Bourke et al., 2014; De Wolf et al., 2024; Newson & Abayomi, 2025).

Interestingly, none of the included studies adopted a proactive or preventive approach that started before weight gain occurred. This points to a reactive pattern where weight-related interventions are typically introduced only after physical changes become clinically clear. Such an approach risks overlooking opportunities for early prevention, particularly at the start of antipsychotic treatment when metabolic risk is highest (Correll et al., 2009). The absence of a systematic focus on physical health might reflect a broader dichotomy in psychiatric care, where the body is often neglected in favor of a narrow focus on mental symptoms (De Hert et al., 2011; Happell et al., 2011). Future interventions should strive for integrated models that address both physical and mental health from the outset.

A prominent barrier to achieving treatment goals in complex, multifaceted areas might be the scarcity of resources in child and adolescent psychiatry (Skokauskas et al., 2019). While nurses frequently incorporate physical healthcare into their routine for individuals with severe mental illness, there remains a need for additional education enhancing effectiveness in this type of care (Kurtoğlu & İnce, 2023). Our findings suggest that a lack of knowledge, recommendations, and guidelines, coupled with the fear of inducing stigma (Bourassa et al., 2017; Jachyra et al., 2018) and/or harming the therapeutic relationship (Jachyra et al., 2018), often inhibits the individual healthcare professionals from addressing weight issues. As such, more research and development of evidence-based tools is urgently needed to prevent healthcare professionals from navigating this issue without clear guidance, increasing the risk that their approach and any advice provided may be shaped by inadequate knowledge and inherent biases.

### 4.1. Strengths and Limitations

The findings of the current review are based on a small number of studies, including participant overlap between two of the studies, and with all studies conducted in the United States and Canada.

As in any literature-based review, there is a possibility that relevant unpublished or grey literature was missed, even though we made efforts to include relevant grey literature through supplementary searches. Including only studies published in English and Scandinavian languages may have limited the perspective and hindered a more global understanding of the issue. By focusing on non-pharmacological studies, this review does not account for emerging treatments such as GLP-1 receptor agonists, which may become increasingly relevant. Including evidence drawn from well-documented and peer-reviewed sources is a strength. Using JBI guidelines and the PRISMA-ScR framework provides a robust methodological foundation, ensuring a comprehensive and transparent approach to the literature search and review process. This adherence to established guidelines enhances the reliability and validity of the findings.

### 4.2. Recommendations for Future Research and Clinical Practice

Addressing overweight and obesity in CAMIs in clinical practice remains an under-acknowledged and insufficiently integrated component of routine mental health care, and there is a pressing need for further investigation to prevent exacerbating negative and longer-term consequences for patients and their families. Our scoping review highlights the need to strengthen research in this emerging area through studies in other countries and continents, and as the field develops, other types of reviews and analyses will be important to compare interventions and methods.

This includes the development and evaluation of integrated or multidisciplinary interventions, studies targeting a broader range of psychiatric diagnoses beyond ADHD and ADD, and longitudinal research assessing both physical and mental health outcomes. More research is needed to investigate the specific needs of families in this context, as well as how best to support and guide children, adolescents, and their parents who are affected by weight gain as a side effect of psychotropic medication. Finally, beyond adopting a person-centered perspective that recognizes the link between mental and physical health and how this affects the individuals in their daily life, professionals in child and adolescent psychiatry should consider the family context and the resources available to them. Rather than focusing primarily on causes, greater emphasis on identifying practical, solution-focused strategies may enhance engagement and outcomes (Patton et al., 2016). Clinicians can also support systemic improvements by advocating for better referral pathways, integrated care models, and policies that enable sustained, coordinated support for young people and their families (World Health Organization, 2023).

## 5. Conclusions

In this review, we found that overweight and obesity in CAMIs appears to be predominantly addressed through an individualistic approach to health and health behavior, potentially impeding comprehensive health promotion and posing risks to the mental and physical well-being of the affected children and adolescents. The need for guidelines on how best to address overweight in child and adolescent mental health care is substantial. More research into the development and implementation of a person-centered and systemic approach that integrates both mental and physical health is warranted. 

## Figures and Tables

**Table 1:** Inclusion and exclusion criteria.

	Inclusion Criteria	Exclusion Criteria
Population	Children and adolescents aged 0–19 years with a diagnosed mental illness (e.g., Attention Deficit/Hyperactivity Disorder (ADHD), anxiety, bipolar disorder, depression, or psychosis)	Patients diagnosed with eating disorders
Concept	Non-pharmacological approaches to addressing overweight or obesity (e.g., lifestyle interventions, behavioral programs, psychoeducation, or service-level strategies)	
Context	Child and adolescent mental health care settings, including specialist services, outpatient clinics, or other clinical or community-based mental health care environments	
Study design	Randomized controlled trials, observational studies, case series, qualitative studies.	Conference abstracts, editorials, commentary articles, discussion paper, theoretical papers, book chapters and reviews (reference lists hand-searched)
Time period	The search was completed in May 2025	
Language	Studies written in English or Scandinavian languages (e.g., Norwegian, Swedish, Danish).	

**Table 2:** Overview of categories and codes.

Categories	Codes
Approaches to weight and health	A one-sided view of weight and health (Bourassa et al., 2017; Dettmer et al., 2021; Espinoza et al., 2021; Jachyra et al., 2018, 2019; Killian et al., 2022; Taylor et al., 2019; Walker et al., 2020; Wykes et al., 2022) Weight and body size are not that important (Jachyra et al., 2019; Walker et al., 2020) The responsibility of weight gain is placed on the individual (Bourassa et al., 2017; Dettmer et al., 2021; Jachyra et al., 2018, 2019; Killian et al., 2022; Taylor et al., 2019; Walker et al., 2020; Wykes et al., 2022) Emphasis on psychosocial factors for enhanced health and well-being (Bourassa et al., 2017; Dettmer et al., 2021; Espinoza et al., 2021; Jachyra et al., 2018, 2019; Killian et al., 2022; Taylor et al., 2019; Walker et al., 2020; Wykes et al., 2022)
Roles, resources and prioritization	Balancing mental illness, symptom reduction and physical health consequences (Bourassa et al., 2017; Jachyra et al., 2018) Lack of time, knowledge and skills (Bourassa et al., 2017; Jachyra et al., 2018; Wykes et al., 2022) Family and HCPs as key influencers (Bourassa et al., 2017; Espinoza et al., 2021; Jachyra et al., 2018; Killian et al., 2022) The better the access, the healthier the choices (Bourassa et al., 2017; Espinoza et al., 2021) Challenges in family involvement (Dettmer et al., 2021; Espinoza et al., 2021; Taylor et al., 2019)
Critical repercussions and future directions	Apprehensions about offending or inducing stigma (Bourassa et al., 2017; Jachyra et al., 2018; Wykes et al., 2022) Stigma permeates (Jachyra et al., 2018, 2019; Taylor et al., 2019) Higher weight is linked to feelings of shame (Jachyra et al., 2018, 2019) Everybody feels uncomfortable in weight-related conversations (Jachyra et al., 2018, 2019; Walker et al., 2020) Aligned expectations (Bourassa et al., 2017; Jachyra et al., 2019)

## Data Availability

This review did not involve the generation of new datasets, and data sharing is therefore not relevant for this manuscript.

## Supplementary Materials

The following supporting information can be downloaded at: https://www.mdpi.com/article/10.3390/bs16010056/s1, Preferred Reporting Items for Systematic reviews and Meta-Analyses extension for Scoping Reviews (PRISMA-ScR) Checklist.

## Abbreviations

The following abbreviations are used in this manuscript:

CAMIs	Children and adolescents with mental illness
HCPs	Health care professionals
ADHD	Attention Deficit Hyperactivity Disorder
ADD	Attention Deficit Disorder
PTSD	Post Traumatic Stress Disorder

## Appendix A

**Table A1:** Data Extraction Chart with Characteristics of Studies.

Author & Year	Country	Aim	Data Collection	Study Population	Inclusion Criteria or BMI	Setting and Diagnoses Represented	Medication	Intervention	Outcome Measures
(Bourassa et al., 2017)	USA	To examine barriers and facilitators of obesity management	Semi-structured interviews	56 participants, including 21 children and adolescents (15 male, 6 female), aged 8–17 years, 20 parents or guardians, and 15 community mental health providers	Overweight/obese or experiencing weight gain since starting a psychotropic medication	Regional community mental health centers ADHD and mood disorder (not otherwise specified)	18 youths were prescribed a second-generation antipsychotic medication		
(Jachyra et al., 2018)	Canada	To examine the perspectives and experiences of children with ASD, their caregivers, and HCPs around discussing weight-related topics in healthcare consultations	Semi-structured, in-depth interviews	21 participants, including 8 * children and adolescents (4 male, 4 female) aged 11–17 years, 8 caregivers (6 mothers and 2 fathers), and five HCPs (2 pediatricians, 2 nurses, and 1 neurologist)	BMI greater than the 85th percentile	Children’s rehabilitation hospital Autism Spectrum Disorder	All the children had allegedly been prescribed psychotropic medication		
(Jachyra et al., 2019)	Canada	To explore children’s perspectives and experiences of discussing weight-related topics in healthcare consultations	Semi-structured interviews	8 * children and adolescents (4 male, 4 female) aged 11–17 years.	BMI greater than the 85th percentile	Children’s rehabilitation hospital Autism Spectrum Disorder	Apri 28: 1 Melatonin: 2 Apo-Quetiapine: 1 Senokot: 1 Abilify: 5 Topiramate: 1 Concerta: 1 Clonidine: 2 Zopiclone: 1 Celexa: 1 Biphentin: 1 Vyvanse: 1 Sertraline: 1 Fluvoxamine: 1 Oxytocin: 1		
(Walker et al., 2020)	Canada	To explore what matters to children with ASD regarding their weight and bodies.	Semi-structured interviews	8 children and adolescents (5 male, 3 female) aged 10–18—median age of 13.5 years.	BMI greater than the 85th percentile	Psychopharmacology clinic at a pediatric teaching hospital Autism Spectrum Disorder	All participants had been prescribed psychotropic medication		
(Taylor et al., 2019)	USA	To discuss the development of a medically supervised inpatient weight reduction program to reduce body mass and improve comorbidities and to evaluate the effectiveness of this program	Retrospective chart review	18 children and adolescents (6 male, 12 female) aged 4–18—mean age of 13 years.	BMI > 99th percentile for age and gender	Children’s Hospital Intellectual disability, depression, bipolar disorder, anxiety, and ADHD.	Not specified	Inpatient treatment Weight management program highly structured to allow for therapy sessions, regular mealtimes, school and reduced sedentary time.	Percent weight loss and percent decrease in BMI from the time of admission to the time of discharge. Secondary: Effect of weight loss on the presence of comorbidities.
(Dettmer et al., 2021)	Canada	To develop and assess a pilot treatment program for youth with The day hospital model served both individuals with severe obesity (obesity group) or those at risk of severe obesity, and individuals with significant mental health issues (psychiatry group).	Anthropometric measures and questionnaires pre- and posttreatment	32 children and adolescents (10 male, 22 female) aged 12–17 years with a mean age of 14.9 years.	11 had primary severe obesity (BMI > 99%) and comorbid mental health conditions (obesity group) and 21 had primary mental illness and were at risk for obesity (psychiatry group).	Psychiatric day hospital at a pediatric quaternary care hospital Generalized anxiety, social anxiety disorder, depression/depressive disorders, ADHD/ADD, OCD, schizophrenia spectrum & other psychotic disorders, disordered eating/binge eating disorders, learning disability/disorder, PTSD/acute stress reaction, trauma/sexual assault, and “other.”	Not specified	Day hospital model Cognitive-Behavioral Therapy-based program that included dialectical behavior therapy, mindfulness, and distress tolerance skills.	Height, weight, BMI, fasting lipid profile, insulin, glucose, and hemoglobin A1c. Eating Disorder Examination Questionnaire (EDE-Q). Secondary: Children’s Depression Inventory 2, Multidimensional Anxiety Scale for Children 2, Pediatric Quality of Life Teen Report 4.0, Peds QL—Healthcare Satisfaction Questionnaire, and the Stress Index of Parenting Adolescents
(Espinoza et al., 2021)	USA	To evaluate participation and weight outcomes in a weight management program that includes a diverse group of participants.	Secondary analysis	20 children (15 male, 5 female) aged 7–13 from 15 different families. 11 families completed the program	BMI ≥ 85th percentile for age and gender	A federally qualified health center in a General Pediatrics clinic Autism Spectrum Disorder	Not specified	A moderate-intensity, manualized Comprehensive Behavioral Family-Based Lifestyle Intervention (CBFLI)	Changes in BMI Z-scores and percentage of the 95th percentile among children with and without ASD. Secondary: Completion rates among children with ASD compared to those without ASD.
(Killian et al., 2022)	USA	To understand the relationship among factors in children, such as sleep duration, BMI, and metabolic parameters.	Baseline extraction from the electronical medical record, regular height and weight measurements during clinical visits, and a baseline questionnaire completed by parents at the initiation of the program.	74 children and adolescents (59 male, 15 female) aged 2–18 years—average of age 11.66 years. 48 patients remained enrolled or attended their 12-month follow-up appointment	BMI ≥ 95th percentile based on age and gender norms	Special Needs Weight Management Clinic Autism Spectrum Disorder and ADHD	18 were prescribed atypical antipsychotics 18, and 20 were prescribed stimulants	Outpatient treatment A multidisciplinary weight management treatment program Clinic recommendations entailed a combination of dietary (MyPlate, decrease sugared drinks, change snacks, increase protein), environmental/behavioral (stimulus control, meal/snack schedules, sleep hygiene), and medical (diagnose/treat sleep apnea, optimize ADHD care, initiate medication management)	Change in BMI percentile 95th score from baseline to 12-month follow-up.
(Wykes et al., 2022)	USA	To characterize community mental health providers’ engagement with youth who have serious emotional disturbances and overweight or obesity, and to identify key predictors of providers’ intentions to involve this group in weight loss programs.	A 41-item questionnaire based on the theory of planned behavior, and a 26-item survey that collected information about engagement in weight-related treatment activities.	101 community mental health providers with an average of 9.3 years of experience in practice	Mental health providers aged 18 years or older who were employed at centres that offered mental health treatment for children, adolescents, young adults, or adults, provided crisis or emergency services, operated in an outpatient setting, delivered specialised care for individuals with SED, and maintained internet-based contact options for managing study materials.	Mental health centers Serious emotional disturbance	Not specified		

* The same 8 adolescents are involved in both studies by (Jachyra et al., 2018, 2019).
